# Identifying latent profiles of child abuse and their gendered effect on adolescent mental health: a school-based study from Rio de Janeiro, Brazil

**DOI:** 10.1590/0102-311XEN185425

**Published:** 2026-06-26

**Authors:** Daniela Porto Faus, Claudia Leite de Moraes, Michael Eduardo Reichenheim, Marcia C. Castro, Claudia Reis Miliauskas, Emanuele Souza Marques, Stella Regina Taquette

**Affiliations:** 1 Universidade Federal do Rio de Janeiro, Rio de Janeiro, Brasil.; 2 Universidade do Estado do Rio de Janeiro, Rio de Janeiro, Brasil.; 3 Harvard TH Chan School of Public Health, Boston, U.S.A.

**Keywords:** Child Abuse, Mental Health, Adolescence, Latent Class Analysis, Maus-Tratos Infantis, Saúde Mental, Adolescência, Análise de Classes Latentes, Maltrato a los Niños, Salud Mental, Adolescencia, Análisis de Clases Latentes

## Abstract

This study aimed to identify patterns of child abuse and neglect and to examine the associations between these patterns and psychological distress during adolescence. A school-based survey was conducted with 693 high school students in Rio de Janeiro, Brazil, during 2016-2017. Latent class analysis was used to identify child abuse and neglect patterns based on the *Childhood Trauma Questionnaire* items’ combination. Psychological distress was measured using the *General Health Questionnaire* (GHQ-12). Multiple linear regression models were employed to examine the associations between child abuse and neglect patterns and psychological distress. Three classes of child abuse and neglect were identified for both sexes: (1) mild emotional violence and physical punishment, (2) emotional maltreatment, physical punishment, and neglect, and (3) poly-victimization. Being classified within the poly-victimization was significantly associated with higher psychological distress in both boys and girls. The class characterized by emotional maltreatment, physical punishment, and neglect was significantly associated with increased psychological distress among girls only. Health and educational services should assess child abuse and neglect when working with adolescents presenting psychological distress, as many may experience multiple forms of victimization. The high prevalence of childhood poly-victimization classes and their strong association with psychological distress highlight the urgent need for intersectoral strategies aiming at reducing child abuse and neglect.

## Abstract (pt)

Este estudo teve como objetivo identificar padrões de abuso e negligência infantil e examinar as associações entre esses padrões e o sofrimento psicológico durante a adolescência. Um inquérito de base escolar foi conduzido com 693 estudantes do Ensino Médio no Rio de Janeiro, Brasil, entre 2016 e 2017. A análise de classes latentes foi utilizada para identificar padrões de abuso e negligência infantil com base na combinação de itens do *Questionário sobre Traumas na Infância*. O sofrimento psicológico foi aferido por meio do *Questionário de Saúde Geral* (GHQ-12). Modelos de regressão linear múltipla foram empregados para examinar as associações entre os padrões de abuso e negligência infantil e o sofrimento psicológico. Três classes de abuso e negligência infantil foram identificadas para ambos os sexos: (1) violência emocional leve e punição física; (2) maus-tratos emocionais, punição física e negligência; e (3) polivitimização. Pertencer à classe de polivitimização associou-se significativamente a um maior sofrimento psicológico, tanto em meninos quanto em meninas. A classe caracterizada por maus-tratos emocionais, punição física e negligência associou-se de forma significativa ao aumento do sofrimento psicológico apenas entre as meninas. Os serviços de saúde e de educação devem rastrear o abuso e negligência infantil ao atender adolescentes que apresentam sofrimento psicológico, uma vez que muitos podem vivenciar múltiplas formas de vitimização. A alta prevalência da classe de polivitimização na infância e sua forte associação com o sofrimento psicológico evidenciam a necessidade urgente de estratégias intersetoriais voltadas à mitigação do abuso e negligência infantil.

## Abstract (es)

El objetivo de este estudio era identificar patrones de abuso y negligencia infantil y examinar las asociaciones entre estos patrones y el malestar psicológico durante la adolescencia. Se realizó una encuesta escolar a 693 estudiantes de secundaria en Rio de Janeiro, Brasil, durante 2016-2017. Se utilizó el análisis de clases latentes para identificar patrones de abuso y negligencia infantil basados en la combinación de los ítems del *Cuestionario de Trauma Infantil*. El malestar psicológico se midió utilizando el *Cuestionario General de Salud* (GHQ-12). Se emplearon modelos de regresión lineal múltiple para examinar las asociaciones entre los patrones de abuso y negligencia infantil y el malestar psicológico. Se identificaron tres clases de abuso y negligencia infantil para ambos sexos: (1) violencia emocional leve y castigo físico, (2) maltrato emocional, castigo físico y negligencia, y (3) polivictimización. La pertenencia a la clase de polivictimización se asoció significativamente con un mayor malestar psicológico tanto en niños como en niñas. La clase caracterizada por maltrato emocional, castigo físico y negligencia se asoció significativamente con un aumento del malestar psicológico solo entre las niñas. Los servicios sanitarios y educativos deberían evaluar el abuso y negligencia infantil cuando trabajen con adolescentes que presenten malestar psicológico, ya que muchos pueden sufrir múltiples formas de victimización. La alta prevalencia de las clases de polivictimización infantil y su fuerte asociación con el malestar psicológico ponen de relieve la urgente necesidad de estrategias intersectoriales destinadas a reducir el abuso y negligencia infantil.

## Introduction

According to the World Health Organization (WHO), approximately one in seven individuals aged 10-19 struggles with mental health problems, many of which remain undiagnosed and untreated ^1^. Mental illness during adolescence significantly impacts growth, development, and overall health, drastically reducing an individual’s quality of life ^2^. Addressing mental disorders, particularly in low- and middle-income countries, involves identifying modifiable risk and protective factors to be incorporated into actions to promote mental health ^3^ ^,^ ^4^. Among the individual modifiable risk factors for mental illness, child abuse and neglect and other childhood adversities are particularly prominent ^5^ ^,^ ^6^ ^,^ ^7^.

The association between child abuse and neglect and mental health has been a subject of sustained inquiry, yielding consistent findings that underscore the heightened vulnerability to various mental health problems following childhood victimization ^6^ ^,^ ^7^. Notwithstanding current evidence, controversies persist regarding the differential impact of various patterns of child abuse and neglect cooccurrence, as well as gender differences. Some epidemiologic studies have suggested that the consequences of child abuse and neglect are similar among men and women ^6^ ^,^ ^7^ ^,^ ^8^. A meta-analysis focusing on child abuse and neglect as a risk factor for depression and anxiety in adults, for example, also suggested that while the effects may be more prevalent in women, this gender difference is not attain statistically significant ^9^. Among adolescents, the impact of child abuse and neglect seems to vary according to the specific mental health outcome under investigation. A study involving 23,395 adolescents in Canada ^10^ suggested that sex did not modify the relationships between any type of child abuse and neglect and internalizing mental disorders. However, Ferreira et al. ^11^ identified a differential effect when analyzing the relationship between physical abuse and alcohol misuse among adolescents in Rio de Janeiro, Brazil, using path analysis.

Some authors argue that the controversies arise from analytical approaches that fail to consider the simultaneous occurrence of different types of child abuse and neglect and its related psychopathological paths ^12^. In line with this, some researchers have pointed out that different child abuse and neglect types overlapping can give rise to unique combinations that show specific mental health outcomes ^13^ ^,^ ^14^ ^,^ ^15^. To overcome this gap, a method capable of identifying patterns of cooccurrence between different child abuse and neglect would be opportune. Person-centered approaches such as latent class analysis (LCA) may represent a step forward in this direction ^13^.

For mapping studies using this new approach, Debowska et al. ^13^ conducted a systematic review synthesizing findings from 16 studies that employed LCA to identify child abuse and neglect patterns. The author highlights the lack of consensus regarding the number and characteristics of latent classes across the studies, underscoring the need for further research in diverse settings and populations. The same review identified that most studies used descriptive approaches when exploring the relationship between child abuse and neglect patterns and other constructs, including mental illness. Such strategies are appropriate when the primary aim is external validation of the estimated classes but may be insufficient when aiming to identify direct and indirect effects of specific classes on mental health ^13^ ^,^ ^16^.

This study aims to examine patterns of childhood abuse and neglect among adolescents and their association with psychological distress, considering potential sex differences. Based on the cumulative harm framework ^14^, we hypothesized that adolescents in poly-victimization child abuse and neglect subgroups would be at greater risk for mental health problems than those in non-victimized or single-form abuse subgroups, and that the child abuse and neglect pattern and its relationship with mental health symptoms would differ between boys and girls.

## Methods

### Study setting

This survey involved second-year high school students attending public and private schools in the IX Administrative Region (IX-AR) of Rio de Janeiro. The IX-AR is a densely populated urban area encompassing 190,000 inhabitants from highly diverse socioeconomic backgrounds. Housing conditions vary widely, from upmarket properties to poorly serviced slums (*favelas*). We opted to include only second-year students to improve the validity of responses and enhance participation rates. Due to their age, participants were more likely to complete the questionnaire accurately than first-year students. Moreover, they were more willing to participate than final-year students, who are typically experiencing the stress associated with university entrance examinations.

### Sample frame and participants

The study employed a probabilistic multistage stratified sampling procedure. The IX-AR includes five public and 15 private schools, which together offer 29 second-year high school classes in public schools and 23 in private schools, with 476 and 805 students, respectively. This population was first stratified into three groups: private schools with day classes, public schools with day classes, and public schools with evening classes, from which students were sampled. A total of 26 classes (747 students) were selected, with the probability of selection proportional to school size. All students in these classes were invited to participate. Only 28 (3.7%) refused to participate. Of the 719 participants, 693 (96.3%) were adolescents (10-19 years) ^2^, who constituted the final sample analyzed in this paper. Questionnaires with incomplete responses were still considered valid.

### Fieldwork and measurements

Data were collected from September 2016 to February 2017 via a multidimensional self-completion questionnaire administered to students during classroom sessions supervised by a fieldwork coordinator. child abuse and neglect were assessed using the QUESI (from Portuguese *Questionário sobre Traumas na Infância*), the Brazilian cross-culturally adapted version of the *Childhood Trauma Questionnaire* (CTQ) ^17^ ^,^ ^18^. The questionnaire assesses violent experiences up to the age of 10, as reported during adolescence and adulthood. The scale consists of 28 items, divided into five subscales: emotional, physical, and sexual abuse, as well as physical and emotional neglect. Each subscale includes five items, with three extra items added to enhance the assessment process. The response options follow a Likert-type scale with five alternatives: never true, rarely true, sometimes true, often true, and very often true. The items with positive connotations were reversed-scored. In this study, participants who selected options rarely true to very often true were considered positive respondents for the item.

We used the Brazilian version of the *General Health Questionnaire* (GHQ-12) ^19^ to assess mental health status. The GHQ-12 has been widely used to measure psychological distress, defined as a state of emotional suffering that includes symptoms of depression and anxiety ^20^. Adolescents were asked to rate the extent to which they had experienced each of the 12 symptoms in the past two weeks. The items are evenly split between positively worded and negatively worded. For the positive group, response options are more so than usual, same as usual, less than usual, and much less than usual. For the negative items, the alternatives are not at all the same as usual, a little more than usual, and much more than usual. We used the bimodal scoring method (0-0-1-1). Total possible scores ranged from 0 to 12, with higher scores indicating greater psychological distress ^21^.

Race/Skin color was self-classified into five categories, as defined by the Brazilian Institute of Geography and Statistics (IBGE, acronym in Portuguese): White, Mixed-race, Black, Yellow, and Indigenous. The family’s economic condition was measured via the Brazilian Economic Classification Criteria (CCEB, acronym in Portuguese) - 2015 version ^22^. The CCEB define broad economic classes based on ownership of durable goods, domestic employee contracts, educational level of the head of the family, and access to public services. Using a scoring system that weighs the number of items in the household and other relevant characteristics, families are divided into A, B, C, D, and E economic classes, A being the class with the highest and E with the lowest purchasing power. Maternal schooling was measured by years of education. For this study, this variable was categorized into three levels: up to 8 years of schooling (up to middle school), 9 to 15 years (incomplete high school, complete high school, and incomplete higher education), and 16 years or more (higher education). Family composition during childhood was assessed by asking whether the adolescents had lived with both parents until the age of 9 (yes/no). Maternal age at the participant’s birth was calculated by subtracting the adolescent’s age from the mother’s age at the time of data collection.

### Data analysis

#### Identifying child abuse and neglect patterns

LCA was used to identify homogeneous groups (classes) of child abuse and neglect. LCA is an individual-centered approach based on the assumption that associations among a set of observed categorical variables (each item of the QUESI) can be explained by a finite number of mutually exclusive classes ^23^. Models with an increasing number of latent classes were specified in an iterative process to identify the best solution using the robust maximum likelihood estimators ^24^. We selected the latent classes by comparing fit indices of k class to k-1 class model using the Vuong-Lo-Mendell-Rubin (VLMR) test, the Akaike information criterion (AIC), and the Bayesian information criterion (BIC). The Bayes factor (BF) was also used to support decision-making ^23^. Model evaluation indices were used to assess whether the k-class provided a better fit than the k-1 class solution. Entropy was also evaluated, with high values indicating low classification error. Values starting at 0.70 were considered acceptable. We also evaluated the plausibility and interpretability of the latent classes, considering previous evidence when selecting the final model. To label the latent classes, we examined the emerging item profiles as represented by the item response probabilities for each latent class ^23^.

#### Assessing the relations between child abuse and neglect latent classes and mental health

After identifying the best-fitting LCA model, we estimated the posterior probabilities for individuals of each class. Adolescents were assigned deterministically to a specific class based on the maximum posterior probability. To address heteroscedasticity of errors, we used a maximum likelihood estimator with robust standard errors.

To examine the relationship between child abuse and neglect latent classes and the mental health outcome, we specified a directed acyclic graph (DAG) incorporating potential confounding factors identified in literature. We determined the minimal set of confounders necessary for unbiased estimates separately for both sexes using DAGitty software (http://www.dagitty.net/). These variables included maternal schooling, ethnicity/skin color, family composition, and maternal age at the participant’s birth.

For the statistical analysis, multigroup models were conducted using sex as the grouping variable to assess whether the relationship between child abuse and neglect patterns and psychological distress differed between boys and girls ^25^. Since the outcome (GHQ-12 score) has an approximate Gaussian distribution, we used a linear model.

Descriptive and diagnostic analyses were conducted using R, version 4.0.4 (http://www.r-project.org). The *survey* package was used to account for complex sampling procedure ^26^. LCA and multigroup analysis were performed using Mplus, version 8 (https://www.statmodel.com/) ^24^. All analyses accounted for the complex sampling structure of the data.

### Ethics

The Research Ethics Committee of the State University of Rio de Janeiro approved the research protocol (Certificate of Presentation for Ethical Appreciation n. 48107514.2.0000.5282), along with the approval of the Rio de Janeiro State Education Secretariat.

## Results

### Sample profile

The sample comprised a balanced distribution of boys and girls, with a mean age of 16.7 years (not shown in the tables). Most students self-identified themselves as White (52.1%). Most of the participants (54.8%) belonged to the Brazilian middle economic class, with an average monthly household income of BRL 4,427.4 (USD 1,383.6) ^22^ (not shown in the tables); conversion rates were calculated based on the time of data collection. Only 15.3% of adolescents had mothers with eight years of schooling or more. More than a quarter of adolescents (27.2%) had never lived with both parents during childhood or who never lived with both parents, and 10% of participants were born to teenage mothers (Table 1).

**Table 1:** Sociodemographic, family, and school characteristics of the study sample.

Characteristics	n *	% **	95%CI
Demographic profile			
Sex			
Female	316	46.3	46.2-46.3
Male	377	53.7	53.6-53.8
Age (years)			
15-16	281	49.0	48.9-49.0
17	248	32.0	32.0-32.1
18-19	164	19.0	18.9-19.1
Race/Skin color			
White	318	52.1	52.0-52.1
Mixed-race	236	31.0	31.0-31.1
Black	114	14.5	14.5-14.6
Yellow	14	1.6	1.6-1.6
Indigenous	7	0.8	0.8-0.8
Socioeconomic status			
Economic class			
A (more affluent households)	90	17.4	17.3-17.4
B	327	54.8	54.8-54.9
C	209	26.6	26.6-26.7
D-E (poorer households)	11	1.2	1.2-1.2
Maternal education (years of schooling)			
≤ 5	291	39.3	39.2-39.4
6-8	241	45.4	45.3-45.5
> 8	123	15.3	15.3-15.3
Family composition			
Lived with both parents during childhood			
No	176	27.2	27.1-27.2
Yes	517	72.8	72.8-72.9
Mother’s age when the adolescent was born (years)			
≤ 18	81	11.0	11.0-11.1
19-24	228	32.8	32.8-32.9
≥ 25	342	56.2	56.1-56.2

95%CI: 95% confidence interval.

* Raw numbers unweighted; the number of missing values for each variable was calculated as follows: total sample size (N = 693) minus the number of observed responses for that variable;

** Estimates considering sample weights.

### Latent class model selection and description of latent profiles

We examined models with 2 to 6 latent classes, separately for girls and boys. The three-class solution showed the best fit (Table 2) and provided the most straightforward theoretical interpretation for both subgroups. The models also showed high entropy (> 0.80), indicating a good level of accuracy in classifying individuals and distinguishing between classes.

**Table 2:** Fit indices for the latent class analysis of child abuse and neglect for girls and boys.

Model	AIC	BIC	BF *	VLMR	Entropy
Girls					
2 classes	7526.331	7726.740	1.2277E+16	0.0390	0.878
3 classes	7350.068	7652.647	0.000111889	0.4313	0.848
4 classes	7266.095	7670.843	5.61258E-06	0.7561	0.860
5 classes	7188.107	7695.024	4.65383E-14	0.5581	0.881
6 classes	7147.335	7756.421	-	0.6239	0.902
Boys					
2 classes	6092.244	6283.625	11467184.59	0.1657	0.839
3 classes	5962.167	6251.115	0.068700418	0.6257	0.825
4 classes	5869.956	6256.471	4.95145E-08	0.3180	0.863
5 classes	5806.031	6290.113	5.2459E-17	0.5640	0.907
6 classes	5783.437	6365.086	-	0.7855	0.903

AIC: Akaike information criterion; BIC: Bayesian information criterion; BF: Bayes factor; VLMR: Vuong-Lo-Mendell-Rubin likelihood ratio test.

* Relationship between k classes and k+1 classes.

**Figure 1:**
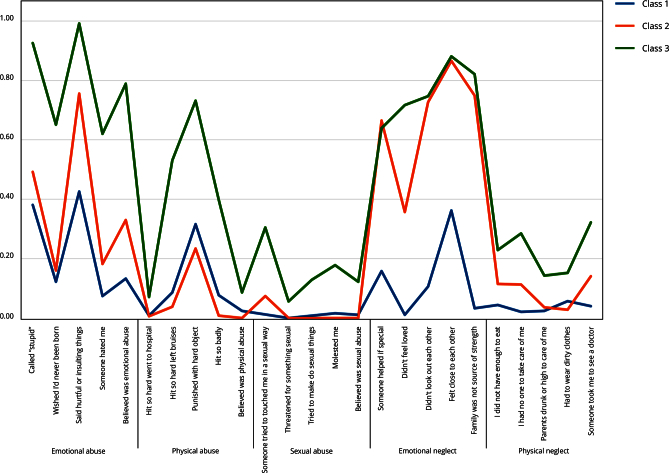
Latent profile plot of child abuse and neglect among girls.

Note: class 1 = 40%; class 2 = 28%; class 3 = 32%.

**Figure 2:**
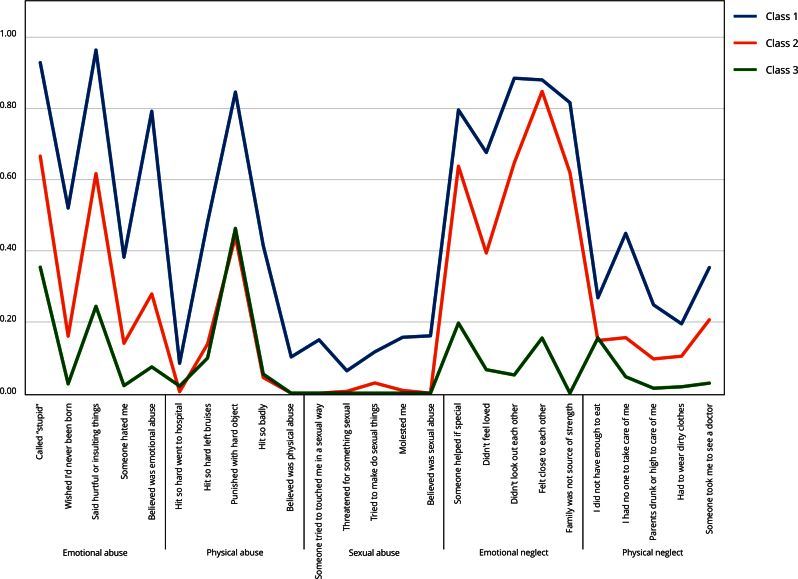
Latent profile plot of child abuse and neglect among boys.

Note: class 1 = 35%; class 2 = 46%; class 3 = 19%.

Figures 1 and 2 show latent item profile plots for girls and boys, respectively. The plots display the per-class probabilities of endorsing each child abuse and neglect item. The estimated classes were similar for both sexes and labeled accordingly. The first latent class was characterized by low to moderate endorsements of emotional violence items and one item from the physical subscale related to physical punishment. This class was labeled “mild emotional violence and physical punishment”. The second class showed a high endorsement of emotional and physical punishment and neglect items, and was thus labeled “emotional maltreatment, physical punishment, and neglect”. The third class included those jointly experiencing all forms of violence (emotional, physical, and sexual) as well as neglect (emotional and physical) and was called “poly-victimization class”.

The prevalence of each class by gender is shown at the bottom of both figures. The prevalence in class 1 is similar for boys and girls (35% and 40%, respectively), but there is a nearly 70% greater proportion of girls in class 3 (poly-victimization class) compared to boys. A closer examination of the frequency of endorsement for various items reveals that girls more frequently report experiences of emotional abuse and neglect in classes 1 and 2, and sexual abuse in class 3. In contrast, boys are more likely to endorse instances of physical abuse, particularly in classes 2 and 3, as well as physical neglect in the latter class.

### The relations between child abuse and neglect patterns and psychological distress

The linear regression coefficients indicated that more severe and cumulative child abuse and neglect classes significantly increased the GHQ-12 scores for both sexes (Table 3). Being in the “poly-victimization” class raised the GHQ-12 scores by approximately 2.7 and 3.5 points compared to those in the “mild emotional violence and physical punishment” class for girls and boys, respectively. The single-parameter model was the most parsimonious for girls, showing that the score difference from class 3 is twice that from class 2, relative to the reference class. This reflects a linear dose-response relationship between child abuse and neglect cooccurrence and the severity of psychological distress. For boys, the increase observed from class 1 to class 2 among boys, although small, was statistically significant. The score difference from class 3 to the reference class is more than double that of class 2, further highlighting the significant impact of poly-victimization on mental health in this subgroup.

**Table 3:** Relationship between latent classes of child abuse and neglect and mental health among adolescents.

Latent classes by sex stratum	β *	95%CI
Girls		
Class 1: mild emotional violence and physical punishment	Reference	
Class 2: emotional maltreatment and physical punishment	1.36	1.01-1.78
Class 3: poly-victimization	2.72	2.02-3.56
Boys		
Class 1: mild emotional violence and physical punishment	Reference	
Class 2: emotional maltreatment and physical punishment	0.67	0.16-1.17
Class 3: poly-victimization	3.45	2.40-4.51

95%CI: 95% confidence interval.

* Regression coefficients, adjusted for maternal education, race/skin color, family composition, and maternal age when the adolescent was born.

## Discussion

This study identified three latent classes of child abuse and neglect for both boys and girls. The qualitative similarities in the classes’ profiles across sexes justified their standard labels. However, there were discrepancies in item endorsements. Specifically, girls showed a higher endorsement of items related to emotional and sexual abuse and emotional neglect, while boys were more likely to report physical abuse and neglect. Supporting our first hypothesis, which is grounded in the cumulative harm framework, adolescents in the poly-victimization class showed higher GHQ-12 scores compared to those in milder classes. However, the pattern of child abuse and neglect and the impact of belonging to the intermediate violence classes differed between boys and girls. These findings contribute to the ongoing debate highlighted in the introduction regarding whether the consequences of child abuse and neglect vary by sex.

A troubling issue emerges when analyzing the class profiles: even adolescents classified within the least severe violence category (class 1) reported experiences of emotional abuse and physical punishment. Among boys, in this class, the likelihood of endorsing a physical punishment item was even higher than that of endorsing an emotional abuse. Beyond its immediate physical consequences, so-called disciplinary practices involving physical punishment can hinder child development, negatively impact school performance, and contribute to long-term mental health issues ^27^. Beyond these negative consequences, its use as a disciplinary strategy reinforces the notion that violence is an acceptable method of conflict resolution. Not finding a “violence-free” class reflects the pervasiveness of child abuse and neglect among adolescents in this context.

The severity of the situation is further underscored by the fact that most boys (65%) and girls (60%) reported experiencing various forms of violence and were classified into the two most severe child abuse and neglect categories. These findings contrast with previous studies conducted in high-income countries, where most individuals fell into the milder child abuse and neglect classes ^13^ ^,^ ^28^. The high levels of violence observed among adolescents in Rio de Janeiro are likely to have multiple explanations. Study suggests that living in areas with high levels of community violence, a common reality in large Latin American cities, can increase the likelihood of violence within family relationships, particularly between parents and children ^29^. This harmful dynamic has also been linked to negative effects on parental mental health ^30^. Given this context, strategies to mitigate the mental health consequences of child abuse and neglect should extend beyond the child and family environment to encompass broader interventions that address multiple forms of victimization, including community violence. Moreover, the primary prevention of child abuse and neglect should constitute a central focus of public health policies and interventions.

The situation is even more concerning for girls, who were more likely to be classified within the poly-victimization group than boys due to their greater exposure to childhood sexual abuse (32% vs. 19%). Although comparative studies on child abuse and neglect patterns between sexes remain scarce ^13^, some research aligns with our findings by indicating that girls and women are more often classified into groups characterized by sexual abuse ^13^ ^,^ ^31^. Conversely, boys were more likely to be classified within the “emotional maltreatment and physical punishment” class, consistent with their more frequent identification as primary victims of punishment, physical abuse, and neglect (46% vs. 28%).

For both sexes, the key difference between the second and the reference class was driven by an increased likelihood of experiencing emotional abuse and a stronger endorsement of emotional and physical neglect items. The cooccurrence of these forms of maltreatment may heighten the risk of negative mental health outcomes. When inflicted by parents or caregivers, emotional abuse and neglect can leave profound and lasting impacts on personality development ^32^ ^,^ ^33^. These types of abuse also affect brain networks responsible for emotion processing and regulation. Moreover, they impact brain networks involved in self-referential processing, sociocognitive functions, emotion management and regulation, and are associated with impaired recovery of the neuroendocrine response to acute stress ^34^. Emotional abuse is also linked to epigenetic changes in genes that regulate the neuroendocrine stress response system - changes that are implicated in increased mental health risks. Conversely, individual factors such as self-efficacy and emotional stability seem to play a key role in buffering the impact of child abuse and neglect and other adverse childhood experiences (ACEs) on both mental and physical health-related quality of life ^35^. Moreover, violence is recognized as a social determinant of health. This perspective broadens the understanding that its consequences extend beyond biological harm, encompassing symbolic and social dimensions that shape how victims perceive the world, form relationships, and engage socially. In this sense, violence can simultaneously occupy a structural role in the constitution of the subject and the production of illness and function as a contextual factor.

Scrutinizing the profile of adolescents in class 3 revealed a cumulative victimization pattern, encompassing emotional, physical, and sexual abuse. The substantial proportion of girls (32%) and boys (19%) in this group highlights the urgent ^22^ need for prevention strategies, early detection protocols, and immediate, qualified care. Figure 1 underscores the severity of the issue, particularly among girls, by illustrating the high probability of endorsing sexual violence items. The extensive literature on the subject has consistently documented the high prevalence of sexual violence against girls ^27^ ^,^ ^36^. Child sexual abuse leads to immediate adverse outcomes, including sexually transmitted infections, physical trauma, unwanted pregnancy ^37^, and long-term mental health consequences such as severe depression and suicidal tendencies. Given these risks, it is imperative to prioritize comprehensive prevention efforts and intervention strategies ^27^ ^,^ ^38^.

Concerning the effect of child abuse and neglect patterns on mental health, the similarity of the results between boys and girls in the most severe child abuse and neglect class concerning GHQ-12 scores aligns with previous findings, which indicate no evidence of effect modification by sex ^5^ ^,^ ^39^. Specifically, boys in class 3 have a mean score more than three points higher than those in class 1. While the increase is slightly lower for girls, it still approaches three points on the scale. These results are both statistically significant and clinically relevant, as a three-point increase is commonly used as a cutoff for identifying suspected cases of psychological distress ^21^. This increase in scores may be explained by the accumulation hypothesis, which suggests that the greater the number of ACEs, the more significant their negative impact on health ^14^.

Notable sex differences emerged when comparing mental distress (GHQ-12) scores between the class (2) - characterized by high levels of emotional abuse, emotional neglect (among girls), physical neglect (among boys), and physical punishment - and the reference class (1). No significant association was observed among boys. In contrast, girls showed an average 11.4% increase in GHQ-12 scores, indicating a visible link between childhood emotional abuse and psychological distress during adolescence. This difference may be attributed to distinct parenting practices and socialization strategies shaped by sexist cultural norms in Brazil. Boys tend to experience less parental supervision, gain earlier approval to engage with peers, and receive more encouragement to attend parties and engage in other social activities. These experiences may serve as protective factors, helping them to reduce stress and mitigate the impact of adverse childhood experiences on mental health. In contrast, girls often face stricter parental control and are more likely to be reprimanded for displaying aggression. As a result, they are more prone to internalizing distress, leading to higher levels of anxiety and depression ^40^.

Our findings have both limitations and strengths that should be considered when interpreting them. One limitation is the potential for underreporting violence and other sensitive experiences in school surveys. Adolescents may fear retaliation or exposure, leading to reluctance to disclose child abuse, particularly if they feel embarrassed. To mitigate this, we used a validated self-response questionnaire, ensured privacy during completion, and reinforced confidentiality. Another limitation relates to truancy. Adolescents who experience severe abuse may struggle academically and drop out before high school, meaning our study may have missed some of the most affected cases. Similarly, teenagers with severe mental health issues may be more likely to leave school early. The absence of these groups could introduce selection bias due to non-random attrition, potentially affecting the accuracy of our estimates. The fact that data collection occurred before the COVID-19 pandemic (2016-2017) may raise questions regarding the interpretability of our findings and may be considered also a limitation of the study. Similarly, although the COVID-19 pandemic may have increased the overall prevalence of mental health problems, there is no evidence that it altered the underlying relationship between violence exposure and mental health outcomes. Unfortunately, these assumptions cannot be empirically tested with the available data. Finally, note that, as this is a cross-sectional study, causal relationships cannot be completely established - even though the temporal sequence of events (childhood violence and adolescent mental health outcomes) may be suggested otherwise.

Our study also has notable strengths. One important feature was the use of the QUESI to assess trauma occurrences before the age of 10. This instrument is not only well-validated in Brazil, but also enables broader comparisons of results, as it is conceptually related to and informed by the CTQ. Beyond identifying child abuse and neglect experiences, the QUESI explicitly defines a recall period, capturing instances of violence that occurred before the age of 10. While this is a cross-sectional study, this methodological approach helps minimize the risk of reverse causation between child abuse and neglect (exposure) and mental health (outcome). Another strength lies in our use of individual QUESI items rather than aggregated dimension scores to construct the latent classes. This approach enabled a more nuanced analysis, revealing patterns that may have been overlooked if we had relied solely on broader violence categories.

## Conclusion

This study identified three distinct patterns of childhood victimization among adolescents in Rio de Janeiro, revealing that most individuals experienced multiple forms of emotional, physical, and sexual abuse or neglect. While boys faced higher rates of physical punishment and neglect, girls were more frequently victims of sexual abuse and poly-victimization. Adolescents exposed to more severe and cumulative victimization showed higher psychological distress, highlighting the lasting mental health impact of childhood abuse. These findings emphasize the need for targeted prevention and intervention strategies, including routine screening for child abuse history in mental health assessments, particularly for girls, who are more vulnerable to emotional maltreatment and sexual abuse. Strengthening violence prevention laws, promoting positive parenting practices, providing economic support to families, and enhancing children’s emotional regulation and help-seeking skills are also crucial steps. Moreover, expanding access to counseling and trauma care also aligns with global initiatives like the WHO’s INSPIRE program, fostering a safer and more supportive environment for children.

## Data Availability

The research data are available upon request to the corresponding author.
